# Optimizing postbiotic production through solid-state fermentation with *Bacillus amyloliquefaciens* J and *Lactiplantibacillus plantarum* SN4 enhances antibacterial, antioxidant, and anti-inflammatory activities

**DOI:** 10.3389/fmicb.2023.1229952

**Published:** 2023-09-07

**Authors:** Yucui Tong, He'nan Guo, Zaheer Abbas, Jing Zhang, Junyong Wang, Qiang Cheng, Shuyue Peng, Tiantian Yang, Ting Bai, Yichen Zhou, Jinzhuan Li, Xubiao Wei, Dayong Si, Rijun Zhang

**Affiliations:** ^1^Laboratory of Feed Biotechnology, State Key Laboratory of Animal Nutrition, College of Animal Science and Technology, China Agricultural University, Beijing, China; ^2^School of Medicine, Tsinghua University, Beijing, China; ^3^School of Pharmaceutical Sciences, Tsinghua University, Beijing, China

**Keywords:** solid-state fermentation, postbiotics, antibacterial, antioxidant, anti-inflammatory

## Abstract

**Background:**

Postbiotics are an emerging research interest in recent years and are fairly advanced compared to prebiotics and probiotics. The composition and function of postbiotics are closely related to fermentation conditions.

**Methods:**

In this study, we developed a solid-state fermentation preparation method for postbiotics with antimicrobial, antioxidant, and anti-inflammatory activities. The antibacterial activity was improved 3.62 times compared to initial fermentation conditions by using optimization techniques such as single factor experiments, Plackett–Burman design (PBD), steepest ascent method (SAM), and central composite design (CCD) methods. The optimized conditions were carried out with an initial water content of 50% for 8 days at 37°C and fermentation strains of *Bacillus amyloliquefaciens* J and *Lactiplantibacillus plantarum* SN4 at a ratio of 1:1 with a total inoculum size of 8%. The optimized SSF medium content ratios of peptide powder, wheat bran, corn flour, and soybean meal were 4, 37.4, 30, and 28.6%, respectively.

**Results:**

Under these optimized conditions, postbiotics with a concentration of 25 mg/mL showed significant broad-spectrum antibacterial capabilities against *Escherichia coli, Salmonella, and Staphylococcus aureus* and strong antioxidant activity against ABTS, DPPH, and OH radicals. Moreover, the optimized postbiotics exhibited good anti-inflammatory ability for reducing nitric oxide (NO) secretion in RAW 264.7 macrophage cells in response to LPS-induced inflammation. Furthermore, the postbiotics significantly improved intestinal epithelial wound healing capabilities after mechanical injury, such as cell scratches in IPEC-J2 cells (*p* < 0.05).

**Conclusion:**

In brief, we developed postbiotics through optimized solid-state fermentation with potential benefits for gut health. Therefore, our findings suggested that the novel postbiotics could be used as potential functional food products for improving body health.

## 1. Introduction

The gastrointestinal (GI) tract is a complex ecosystem teeming with microorganisms such as bacteria, fungi, archaea, protozoa, and viruses that form the intestinal microbiota. The intestinal microbiota has adopted a remarkable spectrum of vital functions for the host including digestion, nutrient absorption, fermentation of dietary fibers, energy generation, and pathogen defense, due to its close relation and evolution with the intestinal environment (Goto, 2019; Lavelle and Sokol, 2020). Food-borne pathogens and subsequent secondary infections cause GI microbial dysbiosis and its metabolites thus causing severe damage to the intestinal mucosa, leading to recurrent intestinal inflammation and simultaneously leading to a variety of diseases such as inflammatory bowel diseases (IBDs), ulcerative colitis, Crohn's disease, and colorectal cancer (Gut et al., 2018; Cheung et al., 2021; Bai et al., 2022). Recent studies revealed that the gut microbiota and its metabolites play a significant role in promoting gut health by preserving the intestinal barrier and the immunity of the host. These metabolites were named postbiotics and defined as “inanimate microorganisms and/or their components that confer a health benefit on the host” in 2021 by “The International Scientific Association of Probiotics and Prebiotics” (ISAPP) (Salminen et al., 2021). The concept of postbiotics is fairly advanced in comparison with prebiotics and probiotics. Postbiotics are differentiated by their elemental composition such as organic acids, bacteriocins, fatty acids, bioactive peptides, phenolic acid, polysaccharides, or bioactive effects including antimicrobial, antioxidant, anti-inflammatory, and immunomodulatory (Rad et al., 2021b; Blazheva et al., 2022). Postbiotic supplements are not as extensively available yet, but they are superior to probiotics because of their purity, definitive chemical structure, safety profile, long shelf life, mass production capability, precise action, and more targeted responses by specific ligand–receptor interactions (Nataraj et al., 2020; Rad et al., 2021a). Therefore, researchers are using postbiotics to modulate microbial signatures of health, nutrition, and disease status.

Oxidative stress and inflammation are two major factors involved in the progression of a wide variety of chronic human diseases (Kruidenier and Verspaget, 2002; Piechota-Polanczyk and Fichna, 2014). Free radicals, especially oxygen radicals, not only induce oxidative stress but can also interact with pro-inflammatory cytokines in a complex manner. Overproduced pro-inflammatory cytokines may cause excessive inflammation and the deterioration of cardiac and/or renal dysfunction (Rapa et al., 2019). Free radicals such as nitrogen and oxygen radicals are generated by various endogenous systems, exposure to different physiochemical conditions, or pathological states in the body (Silpak et al., 2017). Examples of free radicals include hydroxyl free radicals (OH·), superoxide free radical anion (O${}_{2}^{-}$), nitric oxide (NO·), and peroxyl (RO_2_·). The host body possesses antioxidant protection and repair systems to defend against oxidative damage; these mechanisms are not always effective in preventing all harm (Osseni et al., 2000). Furthermore, antioxidants present in diets may support gut health through the direct involvement in inhibiting oxidative stress in the gut environment and by protecting the microbiota against challenging environmental conditions (Wadanambi et al., 2023). Recent *in vivo* and *in vitro* studies reported that postbiotics have antibacterial, antioxidant, anti-inflammatory, immune-modulatory, and intestinal barrier-regulating capabilities (Aguilar-Toalá et al., 2018; Vallejo-Cordoba et al., 2020). These compounds offer a promising approach to combating food-borne pathogens, reducing oxidative stress and inflammatory response, strengthening the gut barrier, and/or regulating the host immune system (Aggarwal et al., 2022). *Lactiplantibacillus* spp.-derived postbiotics (cell-free supernatants) showed strong antioxidant activity and anti-*Staphylococcus* properties, which were mainly associated with lactic acid and other antibacterial components (Khani et al., 2023). Lactic acid bacteria (LAB) postbiotics also exhibited high antioxidant capacity and total phenolic content; combinations with natural preservatives such as EDTA could reduce food-borne pathogens (Incili et al., 2022). Some postbiotics were even incorporated into bacterial nanocellulose for preparing antibacterial films and were expected to extend food shelf life (Mohammadi et al., 2022).

Functional characteristics and compositions of postbiotics are correlated with the strains, culture medium, and condition under preparation (Chang et al., 2021). Kareem et al. (2014) highlight the importance of fermentation media in influencing the secretion of metabolic products and the biological activities of postbiotics. Solid-state fermentation (SSF) is a traditional fermentation technique used to enhance the nutritional values and functional properties of food processing by-products, with high-end-concentration products that are stable and cost-effective (Hölker et al., 2004; Yafetto, 2022). It can also reduce mutual inhibition between metabolites and increase the yield of active substances, making it a promising method for preparing bioactive postbiotics (Pacularu-Burada et al., 2022). In addition, it is more efficient to enrich the postbiotics with targeted bacteria and optimum conditions (Amiri et al., 2021; Melini et al., 2023). Ooi et al. (2015) reported that the optimization of fermentation media can enhance the inhibitory activity of postbiotics, such as bacteriocins produced by *Lactiplantibacillus plantarum* I-UL4. Furthermore, the antimicrobial activity of postbiotic RS5 was enhanced by 108%, reducing the cost by 85% through their refined medium (Ooi et al., 2021). However, optimizing the fermentation conditions and culture media components usually using the one-factor-at-a-time (OFAT) method is not adequate for investigating the interaction between different variables. This limitation can be overcome using statistical approaches such as Plackett–Burman design (PBD) and response surface methodology (RSM). Pacularu-Burada et al. (2021), using the statistical tools of PBD and RSM, enhanced the postbiotic properties and evidenced differences in the metabolite profiles, thus highlighting its functional potential. Postbiotics are commonly prepared by culturing the LAB in culture media [mainly de Man, Rogosa, and Sharpe (MRS) broth], followed by an extraction step (centrifugation or dialysis and filtration) (Gomez-Sala et al., 2016; Dunand et al., 2019; Barros et al., 2020; Moradi et al., 2021), and other fermentation alternatives such as milk and milk-related media also successfully produced remarkable antifungal activity (Gamier et al., 2019). Most studies revealed the antibacterial or antioxidant functions of postbiotics from submerged fermentation (Rossoni et al., 2020; Kienesberger et al., 2022; Banakar et al., 2023; Khani et al., 2023); however, they barely illustrated the SSF or developed multifunctional postbiotics. Moreover, studies on the preparation of functional postbiotics by solid-state fermentation with LAB and *Bacillus* strains or their combination for the production of potential antibacterial, antioxidant, and anti-inflammatory postbiotics are lacking.

Therefore, this study was designed to obtain postbiotics through SSF using statistically optimized approaches to enhance its antibacterial, antioxidant, and anti-inflammatory potentials, thus improving gut health. Furthermore, this study will highlight the pharmacological capabilities of postbiotics and their safer use in nutrition research in future.

## 2. Materials and methods

### 2.1. Materials, microorganisms, and culture conditions

Peptide powder, wheat bran, soybean meal (not genetically modified), and corn were obtained from Beijing Longgang Biotechnology Research Center (Zhuozhou, Hebei Province, China). For the formulation of the SSF fermentation medium, the corn flour, soybean meal, peptide powder, and wheat bran powder consisted of the basal solid medium, totaling 100% (w/w), together with other ingredients ranging from 0.5 to 1% (w/w) and 2 to 4% (w/w) according to PBD or RSM runs and sterilized at 121°C for 15 min (LS-B100L, Binjiang Medical, China).

*Bacillus amyloliquefaciens* J and *Lactiplantibacillus plantarum* SN4 were selected based on specific functional properties, such as antibacterial activity (Supplementary Figure S1). These strains were possessed by the Laboratory of Feed Biotechnology at China Agriculture University and were deposited at the China General Microbiological Culture Collection Center (CGMCC), Beijing, China. The stock cultures of the *L. plantarum* strain and *Bacillus* strains were reactivated in de Man, Rogosa, and Sharpe (MRS) broth and beef peptone yeast (BPY) broth at 37°C aerobiosis. The cell viability counts of fermentation strains were determined using saline (0.9%, w/v) serial dilutions inoculated in MRS and BPY agar plates. For the established optical density and incubation conditions (12 h, 37°C, aerobiosis), an inoculum size of ca 1 × 10^8^ colony-forming units per milliliter (CFU/mL) was obtained for all fermentation strains.

The pathogen indicator bacteria (*Staphylococcus aureus* ATCC 43300, *S. aureus* ATCC 6385, *Staphylococcus aureus* ATCC 1882, *Staphylococcus aureus* ATCC 25923, *Salmonella typhimurium* ATCC 14028, *Salmonella pullorum* CVCC 519, *Escherichia coli* ATCC 25922, *Escherichia coli* ATCC 25325, Escherichia coli O157:H7 ATCC 43889, *Clostridium perfringens* CVCC 2030, *Pseudomonas aeruginosa* ATCC 27853, and *Pseudomonas aeruginosa* ATCC 9027) used in this study were maintained in our laboratory. All pathogenic bacteria were cultured overnight in Luria–Bertani (LB) broth at 37°C under aerobic conditions.

### 2.2. Determination of the antibacterial activity

The postbiotics were extracted by adding three to five times the weight of sterile water after fermentation, depending on the experiment design, optimization stage, and value of the antibacterial rate. According to the OFAT and PBD experiment designs, postbiotics were diluted three times, and for the RSM experiment design, the dilution was five times for acquiring and distinguishing the difference between runs. Then soaked in an oscillatory for 1 h and centrifuged at 6,000 rpm for 10 min at 4°C, the supernatant was collected and filtered through 0.22 μm filter (Millex-GP, Merck Millipore Ltd., Germany). The microtiter plate-based antibacterial assay was performed according to the method reported by Casey et al. (2004) and Matsue et al. (2019), with slight modifications. To improve screening efficiency in our experiments, the indicator microorganism *E. coli* ATCC 25922 was cultured to the logarithmic growth stage, followed by centrifugation, washing, dilution, and resuspension. Then, 10 μL of bacterial solution with a final concentration of 5 × 10^6^ CFU/mL, sterilized LB medium (140 μL), and test samples (50 μL) were added to the 96-well plates. The wells with only bacterial suspension were used as positive control, and only LB medium was used as test quality control. The plates were incubated for several hours at 37°C in an incubator shaker at 200 rpm (LYZ-D2403, Longyue, China). At the indicated time, the turbidity at OD_600nm_ was measured using a multi-well photometric microplate reader (Spectra Max 190, Molecular Devices, USA). Having established the turbidity values prior to and immediately following incubation, antibacterial activity was calculated using the following Equation (1):


(1) $$\begin{array}{l} \text{Inhibition}\%=\frac{\left({\text{P}}_{12}-{\text{P}}_{0}\right)-\left({\text{T}}_{12}-{\text{T}}_{0}\right)}{\left({\text{P}}_{12}-{\text{P}}_{0}\right)}\times 100 \end{array}$$

where inhibition is the antibacterial ratio (%) of test samples against *E. coli* ATCC 25922, P_12_ – P_0_ is the turbidity change in positive control wells after incubation for 12 h at 37°C, and T_12_ – T_0_ is the turbidity change in the test sample wells under the same condition.

### 2.3. Analytical approach for optimizing culture conditions

#### 2.3.1. One-factor-at-a-time design

One-factor-at-a-time experiments were conducted to explore the influence of different factors, including fermentation strains, temperature, time, inoculum sizes, and water content on antibacterial activity. The values of the main factors in this study were as following: fermentation strains involving single strains, and its combination; temperature set was 28, 31, 34, 37 and 40°C; time was 2, 4, 6, 8, and 10 d; inoculum size was 4, 6, 8, 10, and 12%; and water content was 35, 40, 45, 50 and 60%.

#### 2.3.2. Determination of significant factors using Plackett–Burman design

The PBD was used to identify the key factors of solid medium components that contribute to the antibacterial properties while assuming that the interactions among these factors can be negligible. The corn flour, soybean meal, peptide powder, and wheat bran powder consisted of the basal solid medium, totaling 100%; other components were added extra based on the solid medium. A nine-factor orthogonal matrix was created, with each factor having a high-level (+1) and a low-level (−1) representation. The candidate influent factors were: corn flour (20, 40%) (w/w), soybean meal (15, 30%) (w/w), glucose (4, 8%) (w/w), molasses (2.5, 5%) (w/w), growth factor (0.5, 1%) (w/w), peptide powder (2, 4%) (w/w), CaCO_3_ (0.5, 1%) (w/w), yeast extract, and KH_2_PO_4_ contents (0.2, 0.4%) (w/w), respectively. Correspondingly, (−1, +1) levels were selected for *E. coli* ATCC 25922 inhibition, each experiment reported in the PBD was performed in triplicate (Table 1).

**Table 1:** Plackett–Burman experiments design matrix with factors given in coded levels and inhibition values.

**Run**	**A: Corn flour %**	**B: Soybean meal %**	**C: Glucose %**	**D: Molasses %**	**E: Growth factor %**	**F: Peptide powder %**	**G: CaCO_3_%**	**H: Yeast extract %**	**J: KH_2_PO_4_%**	**Inhibition %**
1	40 (+1)	30 (+1)	4 (−1)	5.0 (+1)	1.0 (+1)	4 (+1)	0.5 (−1)	0.2 (−1)	0.2 (−1)	91.45
2	20 (−1)	30 (+1)	8 (+1)	2.5 (−1)	1.0 (+1)	4 (+1)	1.0 (+1)	0.2 (−1)	0.2 (−1)	53.56
3	40 (+1)	15 (−1)	8 (+1)	5.0 (+1)	0.5 (−1)	4 (+1)	1.0 (+1)	0.4 (+1)	0.2 (−1)	98.14
4	20 (−1)	30 (+1)	4 (−1)	5.0 (+1)	1.0 (+1)	2 (−1)	1.0 (+1)	0.4 (+1)	0.4 (+1)	0
5	20 (−1)	15 (−1)	8 (+1)	2.5 (−1)	1.0 (+1)	4 (+1)	0.5 (−1)	0.4 (+1)	0.4 (+1)	21.69
6	20 (−1)	15 (−1)	4 (−1)	5.0 (+1)	0.5 (−1)	4 (+1)	1.0 (+1)	0.2 (−1)	0.4 (+1)	0
7	40 (+1)	15 (−1)	4 (−1)	2.5 (−1)	1.0 (+1)	2 (−1)	1.0 (+1)	0.4 (+1)	0.2 (−1)	0
8	40 (+1)	30 (+1)	4 (−1)	2.5 (−1)	0.5 (−1)	4 (+1)	0.5 (−1)	0.4 (+1)	0.4 (+1)	55.11
9	40 (+1)	30 (+1)	8 (+1)	2.5 (−1)	0.5 (−1)	2 (−1)	1.0 (+1)	0.2 (−1)	0.4 (+1)	97.77
10	20 (−1)	30 (+1)	8 (+1)	5.0 (+1)	0.5 (−1)	2 (−1)	0.5 (−1)	0.4 (+1)	0.2 (−1)	97.91
11	40 (+1)	15 (−1)	8 (+1)	5.0 (+1)	1.0 (+1)	2 (−1)	0.5 (−1)	0.2 (−1)	0.4 (+1)	97.96
12	20 (−1)	15 (−1)	4 (−1)	2.5 (−1)	0.5 (−1)	2 (−1)	0.5 (−1)	0.2 (−1)	0.2 (−1)	0

#### 2.3.3. Steepest ascent method

The path of the SAM was achieved to set up basal concentrations of media components selected from the PBD to be used in a central composite design (CCD). It permitted rapid movement toward the most favorable of variable concentrations. Increments of X_1_ (glucose), X_2_ (corn flour), and X_3_ (soybean meal) were 2, 5, and 5, respectively. Experiments were performed along with the SAM until the response did not increase anymore, and each experiment was conducted in triplicate. The medium level of factors is represented in Table 2.

**Table 2:** Experimental design and response of the SAM experiments.

**Run**	**X_1_: Glucose (%)**	**X_2_: Corn flour (%)**	**X_3_: Soybean meal (%)**	**Inhibition (%)**
1	4	20	15	0
2	6	25	20	9.79
3	8	30	25	50.6
4	10	35	30	64.21
5	12	40	35	64.09
6	14	45	40	58.34

#### 2.3.4. Central composite design and response surface

Once the critical factors were identified via screening and the experimental design space was approached by SAM, the CCD was used to define the level of the significant parameters and the interactions between them, which significantly influence the antibacterial activity. Each parameter was analyzed at five levels coded as (±α, ±1, 0), as shown in Table 3.

**Table 3:** Response surface of CCD and inhibition of *Escherichia coli* ATCC 25922.

**Run**	**Glucose**		**Corn Flour**		**Soybean meal**		**Inhibition (%)**
	$\bar{{\text{X}}_{1}}$	**X**_1_ **(%)**	$\bar{{\text{X}}_{2}}$	**X**_2_ **(%)**	$\bar{{\text{X}}_{3}}$	**X**_3_ **(%)**	
1	−1	8	−1	30	−1	25	68.37
2	1	12	−1	30	−1	25	47.57
3	−1	8	1	40	−1	25	30.48
4	1	12	1	40	−1	25	26.13
5	−1	8	−1	30	1	35	50.65
6	1	12	−1	30	1	35	30.42
7	−1	8	1	40	1	35	35.36
8	1	12	1	40	1	35	42.95
9	–α	6.64	0	35	0	30	51.88
10	α	13.36	0	35	0	30	41.14
11	0	10	–α	26.59	0	30	68.28
12	0	10	α	43.41	0	30	55.18
13	0	10	0	35	–α	21.59	26.07
14	0	10	0	35	α	38.41	24.55
15	0	10	0	35	0	30	71.19
16	0	10	0	35	0	30	66.37
17	0	10	0	35	0	30	70.13
18	0	10	0	35	0	30	68.79
19	0	10	0	35	0	30	71.46
20	0	10	0	35	0	30	70.32

#### 2.3.5. Regression models and statistical analysis

The experimental data were fitted using Design-Expert 8.0.6 (Stat-Ease, Inc., Minneapolis, MN, USA) and SPSS 26.0 (SPSS Corp., Chicago, IL, USA) software. To determine the inhibition capabilities in relation to input factors according to Table 3, a polynomial regression model was used as follows:


(2) $$\begin{array}{l} {\text{Y}}_{1}=\beta_{0}+\sum_{\text{i = 1}}^{3}\beta_{\text{i}}X_{\text{i}}+\sum_{\text{i < j = 2}}^{3}\beta_{\text{ij}}{\text{X}}_{\text{i}}{\text{X}}_{\text{j}}+\sum_{\text{i = 1}}^{3}\beta_{\text{ii}}{\text{X}}_{\text{i}}^{2} \end{array}$$

where Y_1_ is the antibacterial activity, X_i_ are input variables (three variables retained), and β_0_, β_i_, β_ij_, and β_ii_ are the regression coefficients for the intercept, linear, interaction, and quadratic effects.

In addition, ANOVA was used to assess the statistical variables for the optimization of the fermentation medium, with an F-test used to verify the statistical significance. The coefficient of determination (R^2^) was employed for the quality of the polynomial model equation.

### 2.4. Preparation of postbiotic extracts

After the fermentation with the aforementioned optimized culture conditions, postbiotics were extracted with sterile water and soaked in an oscillatory for 1 h. The extracts were then centrifuged at 6,000 rpm for 10 min, 4°C, and the supernatant collected and filtered through 0.22 μm filter (Millex-GP, Merck Millipore Ltd., Germany). The filtered solutions were freeze-dried by using a lyophilizer (Beijing Fourring Scientific Instrument Co. Ltd., Beijing, China) and stored in a sterile container at −20°C for further research.

### 2.5. Bioactive profile of postbiotics

The profile of postbiotic compositions was detected by various colorimetric and modern instrument analytical methods. The 3-phenyllactic acid (Wu et al., 2020), lactic acid (Russo et al., 2017), and ferulic acid (Kaur et al., 2013) were determined by high-performance liquid chromatography (HPLC), and the content of short-chain fatty acids (SCFAs) including acetic acid, propionic acid, butyric acid, and pentanoic acid was detected by gas chromatography (GC) (Beards et al., 2010). The total protein was measured with BCA Protein Assay Kit (Solarbio) according to the manufacturer's recommendation. Phenolic acid was measured by the Folin–Ciocalteu's phenol reagent (Tan et al., 2021). Total soluble sugar was determined by using the phenol–sulfuric acid method (Nielsen, 2017).

### 2.6. Determination of minimal inhibitory concentrations and minimal bactericidal concentrations

The broth microdilution assay was used to assess the MICs and MBCs, similar to our previous research (Guo et al., 2021), with slight modifications. Sterilized Mueller–Hinton broth (MHB) medium (150 μL) and postbiotic solution (50 μL) were added to sterile 96-well plates at final postbiotic concentrations of 0.4–50 mg/mL. The bacteria were cultured to the logarithmic growth stage, followed by centrifugation, washing, dilution, and resuspension. Then, 2 μL of bacterial solution with a concentration of 5 × 10^6^ CFU/mL was added to the 96-well plates. The wells with only bacterial suspension or MHB medium were used as controls. The minimum postbiotic concentration at which bacterial growth was invisible in the 96-well plate was defined as the MIC of the postbiotics.

The MBCs were tested after determining the MICs. A 20 μL aliquot of culture medium in the wells without visible bacterial growth was spread on MHB agar plates and cultured overnight at 37°C to determine the MBCs based on the absence of bacterial colony growth. Each trial was repeated three times.

### 2.7. *In vitro* antioxidant activity assays

1,1-diphenyl-2-picrylhydrazyl (DPPH) was obtained from Sigma Aldrich. 2,2′-azinobis-3-ethylbenzothiazoline-6-sulphonic acid (ABTS) was obtained from Biotopped life sciences Co., LTD, Beijing, China. Ascorbic acid and salicylic acid were obtained from Solarbio, and other reagents were obtained from Sinopharm Chemical Reagent. The antioxidant activity of hydroxyl radical, DPPH, and ABTS radical scavenging activities were calculated by the flowing formula:


(3) $$\begin{array}{l} \%\text{Scavenging}=1-\frac{\text{A1}-\text{A}}{\text{A0}}\times 100 \end{array}$$

where A_1_ refers to the sample mixed with the working solution, A refers to the sample without the work solution, and A0 is the absorbance of the working solution without the sample as a blank control. Ascorbic acid was used as a positive control.

#### 2.7.1. Hydroxyl radical scavenging activity

The scavenging activity of hydroxyl radicals was detected based on the Fenton reaction of Fe^2+^/H_2_O_2_ (Kaur and Sud, 2023). A 200 μL of the aqueous solutions of different concentrations (0–10 mg/mL) of postbiotics and 300 μL of 1.8 mM FeSO_4_ solution were mixed with 90 μL of 1.6 mM salicylic acid and ethanol solution, and then, 210 μL of 3% (v/v) H_2_O_2_ solution was added. After shaking well, the sample solution was added, and the absorbance was measured at 510 nm.

#### 2.7.2. DPPH radical scavenging activity

The DPPH radical scavenging activity was determined according to the previous method of Wei et al. (2023). In brief, an aliquot of 200 μL of different concentrations (0–10 mg/mL) of postbiotics was added to 800 μL of DPPH solution (100 μM DPPH in absolute methanol). After incubation for 30 min, the absorbance of the incubated samples was measured at 517 nm. Methanol was used as a blank.

#### 2.7.3. ABTS radical scavenging activity

The ABTS radical scavenging was measured according to the method of Sah et al. (2014). A 200 μL of sample solution was added to 600 μL of ABTS radical working (OD_734nm_ = 0.7) solution and incubated in the dark for 30 min, and the absorbance of the mixture at 734 nm was measured. A measure of 200 μL of dd water instead of the sample was used as a blank.

#### 2.7.4. Reducing power

To determine the reducing power, the methods were the same as Kou et al. (2013). The test samples with different concentrations (0–10 mg/mL) of postbiotics (200 μL), 500 μL of sodium phosphate buffer (0.01 M, pH = 6.6), and 500 μL of potassium ferricyanide solutions (1%, w/v) were mixed and incubated the mixtures for 30 min at 50°C. Then, 500 μL of trichloroacetic acid (10%, w/v) was added and centrifuged for 10 min at 3,000 rpm. The upper layer (200 μL) was added with 100 μL of ferric chloride (0.1%, w/v), and the absorbance at 700 nm was measured.

### 2.8. Cell viability assay

The viability of the postbiotic-treated IPEC-J2 cells and RAW264.7 cells was determined using a Cell Counting Kit-8 (CCK-8) assay kit (Solarbio). The IPEC-J2 cells and RAW264.7 cells (3 × 10^4^ cells/mL) were cultured overnight in 100 μL of DMEM in 96-well plates. Postbiotic final concentrations of 0, 2, 4, 6, 8, 10, 12, 14, 16, and 18 mg/mL with cell culture medium were added to IPEC-J2 cells, and final concentrations of 0, 0.5, 1, 1.5, 2, 2.5, 3, 3.5, 4, 4.5, and 5 mg/mL were added in RAW264.7 cells, followed by incubation for 6 h. The medium was replaced with 100 μL of fresh DMEM and 10 μL of CCK-8 solution. After incubation at 37°C for 2–4 h, the absorbance of the plates at 450 nm was determined using a microplate reader. The cell viability was calculated using the following formula:


(4) $$\begin{array}{l} \%\text{Cell viability}=\frac{\text{Abs of samples}}{\text{Abs of control}}\times 100 \end{array}$$

### 2.9. Anti-inflammatory assay

NO is an important inflammatory biomarker responsible for inflammation. We emulated the NO concentration in the RAW 264.7 cell culture supernatants using the Griess reagent according to Han and Hyun (2023). Cultured RAW 264.7 cells (3 × 10^4^ cells/well) were retreated with different concentrations of postbiotics (0–4 mg/mL) and then LPS (100 ng/mL) in 96-well plates and incubated for 24 h. Cell culture supernatants were mixed with an equal volume (50 μL) of the Griess reagent and incubated in 96-well plates for 10 min at room temperature. Absorbance was measured at 540 nm using a microplate reader.

### 2.10. Wound healing assay

To assess wound healing capabilities, IPEC-J2 cells were seeded in 6-well imaging plates at 4 × 10^5^ cells per well and grown overnight to confluency. Selected samples were pretreated with the postbiotics for 6 h, and a straight scratch was generated with constant pressure, speed, and angle using a 100 μL plastic pipette tip and washed by PBS to remove excess debris. For scanning of the scratch-wounded area, images were taken every 12 h by a microscope (DSZ2000). Cell scratch area image analysis was carried out using Image J software.


(5) $$\begin{array}{l} \text{\% Cell healing rate\% Cell healing rate}=\frac{\text{0 h cell scratch area}-\text{n h cell scratch area}}{\text{0 h cell scratch area}}\\ \;\times 100\% \end{array}$$

### 2.11. Statistical analysis

The results were described as mean ± standard error of the mean (SEM). The data were analyzed by one-way ANOVA using an SPSS system. Student Newman–Keuls multiple comparison test was used to determine the difference between treatments. All data were subjected to one-way analysis of variance (ANOVA) with the general linear model, and the significant differences in all of the figures were tested by multiple comparisons and visualized using GraphPad Prism 9 software (Graphpad Software Inc., San Diego, CA). Statistical significance was expressed using *p*-value of < 0.05.

## 3. Results

### 3.1. Effect of fermentation strains, temperature, time, inoculum size, and water content on antibacterial activity of postbiotics

**Figure 1:**
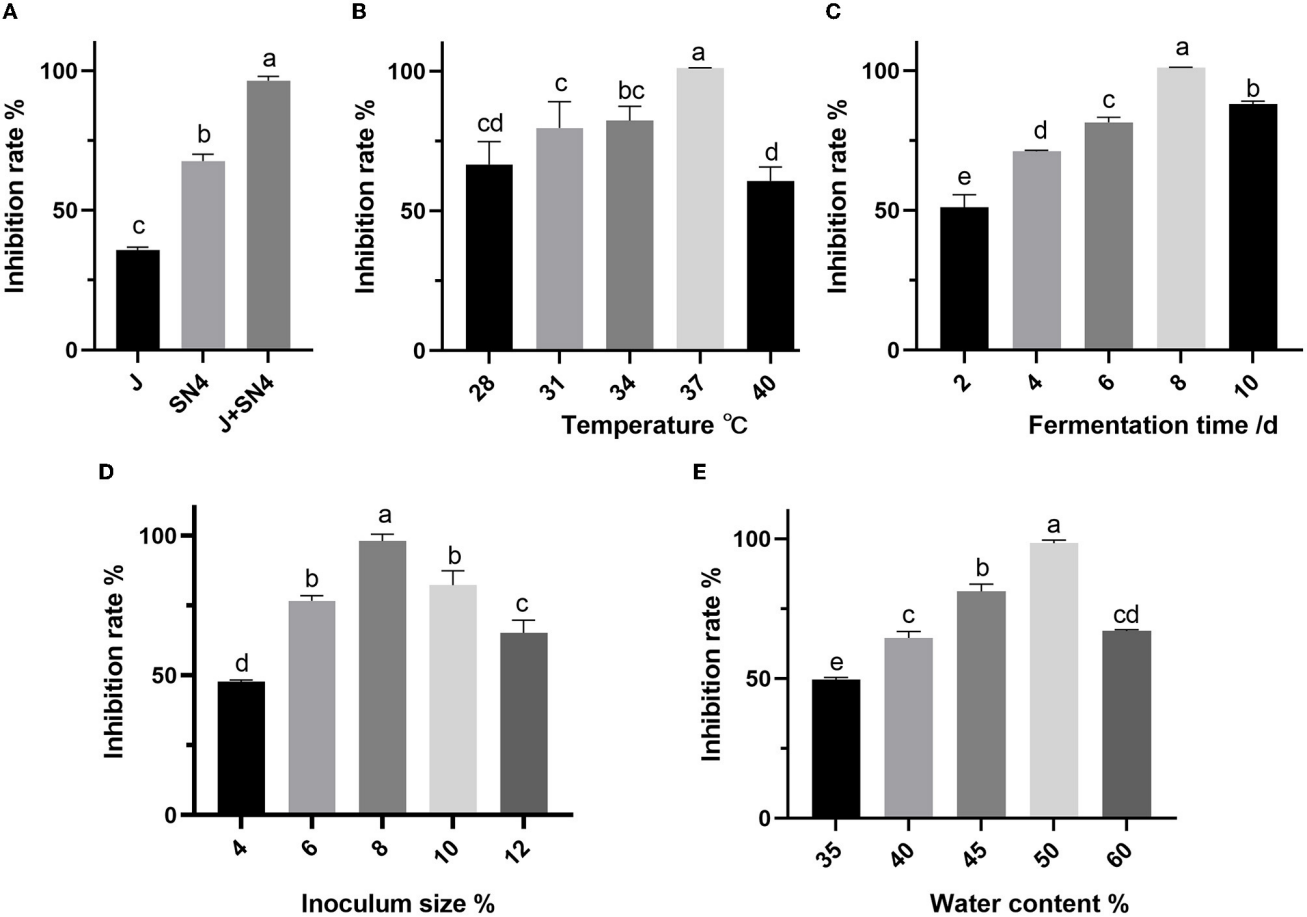
Effect of different fermentation strains **(A)**, temperature **(B)**, time **(C)**, inoculum size **(D)**, and water content **(E)** on the inhibition rate of *Escherichia coli* ATCC 25922. a–e: different lowercase letters represent significance (*p* < 0.05), and *p*-values were expressed as mean ± SEM (*n* = 3).

The antibacterial activity of postbiotics varied depending on the number and combination of fermentation strains, with one to two strains producing different levels of activity as shown in Figure 1. Fermenting with two strains (1:1, v/v) simultaneously was found to be preferable for achieving higher inhibition rates against *E. coli* ATCC 25922 (Figure 1A). Other fermentation conditions with mixed strains fermentation affected by antimicrobial properties are presented in Figure 1. The temperature and water content of fermentation showed a significant influence on inhibition rate, when the temperature was 28–40°C and water content was 35–60%, the antibacterial activity first increased and then decreased as temperature value or water content increased. The inhibition rate achieved the maximum value when the temperature was 37°C and the water content was 50% (Figures 1B, E). The inhibition rate increased significantly from 50 to 100% as fermentation time was extended from 2 to 8 days. With the extended fermentation time to 10 days, the inhibition rate decreased significantly (*p* < 0.05) (Figure 1C). The optimum time was recorded 8 days. Furthermore, a significantly higher inhibition rate (*p* < 0.05) was observed as the inoculum size increased from 4 to 8%, while decreased with the inoculum size of 10–12% (*p* < 0.05). So, the optimized inoculum size was 8%.

### 3.2. Corn flour, soybean meal, and glucose as key factors affecting antibacterial activity

**Figure 2:**
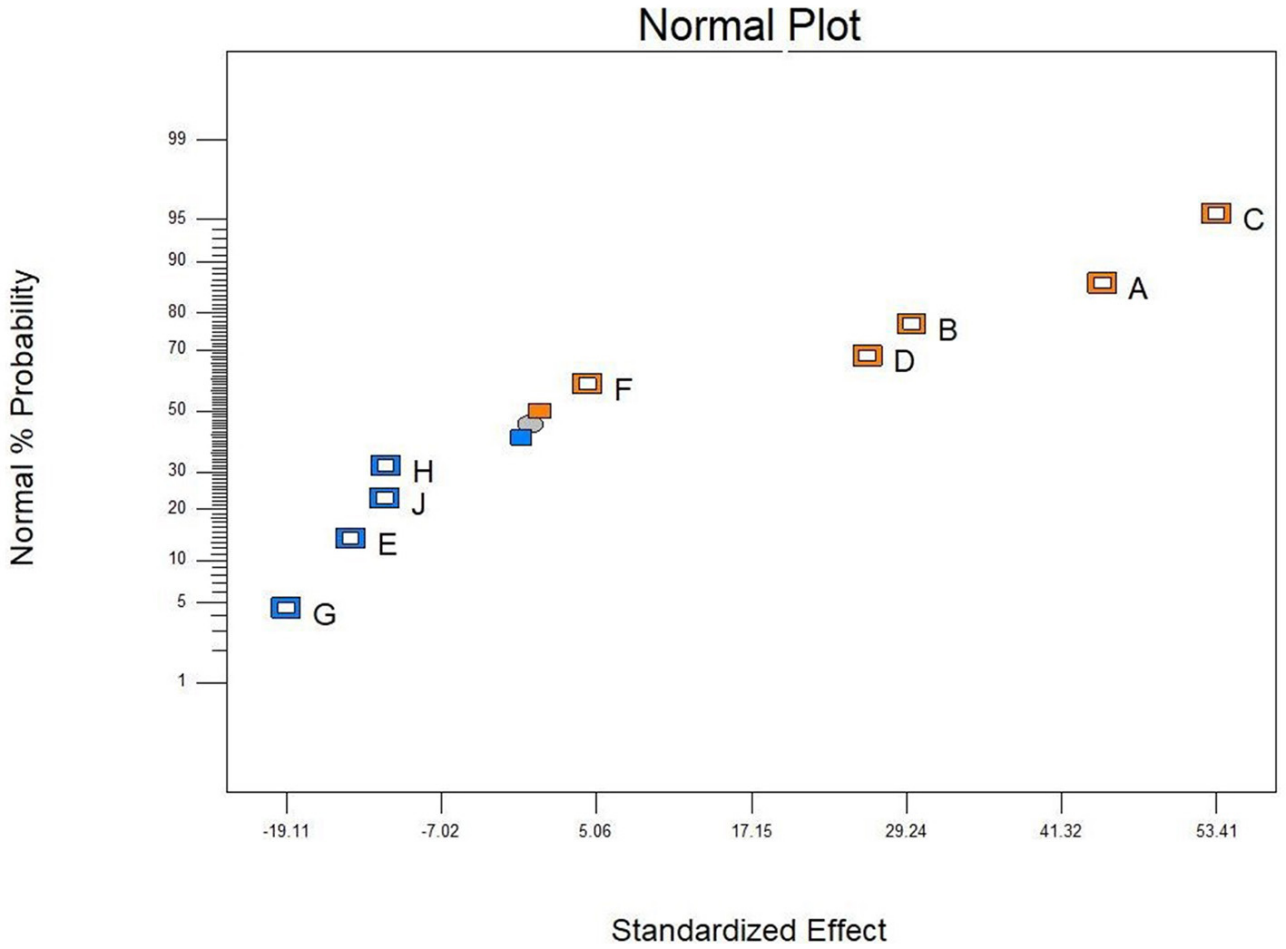
Influence of the factors on the antibacterial activity by PB design. Corn flour **(A)**, soybean meal **(B)**, glucose **(C)**, molasses **(D)**, growth factor **(E)**, peptide powder **(F)**, CaCO_3_ **(G)**, yeast extract **(H)**, and KH_2_PO_4_ **(J)**. Orange block represented positive effect and blue block means negative effect, all factors analyzed by Design-Expert 8.0.6.

The PBD was used to screen the effect of different factors of corn flour, soybean meal, glucose, molasses, growth factor, peptide powder addition, CaCO_3_, yeast extract, and KH_2_PO_4_ on antibacterial activity of postbiotics (Table 1). After analyzing the inhibition rate of postbiotics, corn flour (A), soybean meal (B), and glucose (C) were the main factors that significantly affected the antibacterial activity (*p* < 0.05) (Table 4). According to the three significant factors on antibacterial activity, corn flour (A), soybean meal (B), glucose (C), molasses (D), and peptide powder (F) showed a positive impact, whereas the growth factor (E), CaCO_3_(G), yeast extract (H), and KH_2_PO_4_ (J) had a negative impact (Figure 2). The obtained polynomial model was expressed in terms of coded factors:


(6) $$\begin{array}{l} {\text{Y}}_{1}\%\;=\;-102.38+2.23^{*}\text{A}+1.978^{*}\text{B}+13.35^{*}\text{C}+13.11^{*}\text{D}\\ \;-\;7.02^{*}\text{E}+2.19^{*}\text{F}-9.55^{*}\text{G}-5.66^{*}\text{H}-5.71^{*}\text{J} \end{array}$$

**Table 4:** Statistical analysis of factors using Plackett–Burman design.

**Coefficient**	**Value**	***p*-value**
A	3,897.53	3 × 10^−4^
B	1,729.14	6 × 10^−4^
C	5,604.00	2 × 10^−4^
D	1,350.45	7 × 10^−4^
E	387.47	2.6 × 10^−3^
F	37.75	2.55 × 10^−2^
G	717.18	1.40 × 10^−3^
H	251.54	4.00 × 10^−3^
J	256.22	3.90 × 10^−3^

### 3.3. Optimization by steepest ascent method

The direction of the steepest ascent method was determined by the results of PBD. X_1_ (Glucose), X_2_ (Corn Flour) and X_3_ (Soybean Meal) by incremental steps of 2, 5, and 5, respectively, were added to study the medium component and locate the region of significance, and the results are as shown in Table 2. Glucose, Corn Flour, and Soybean Meal were fixed at 10, 35, and 30%, respectively, and exhibited the highest inhibition against *E. coli* ATCC 25922. This point was chosen as a clue to set up basal concentrations for further optimization by CCD (the center point for optimization by CCD).

### 3.4. Optimization by using CCD

The CCD was used to define the optimum levels of the significant factors and study their interactions. X_1_ (Glucose), X_2_ (Corn Flour), and X_3_ (Soybean Meal) were studied at five levels (–α, −1, 0, 1, α). The experimental design and the experimental responses of inhibitions are reported in Table 3. Based on these data analyzed by Design-Expert 8.0.6, the polynomial regression of predicted responses Y_1_ for inhibition in terms of coded factors is expressed as follows:


(7) $$\begin{array}{l} {\text{Y}}_{\text{1}}=\text{69}.\text{76}-\text{4}.0{\text{9X}}_{\text{1}}-\text{6}.{\text{16X}}_{\text{2}}-\text{1}.{\text{15X}}_{\text{3}}+\text{5}.{\text{53X}}_{\text{1}}{\text{X}}_{\text{2}}\\ \;+\text{1}.{\text{56X}}_{\text{1}}{\text{X}}_{\text{3}}+\text{7}.0{\text{7X}}_{\text{2}}{\text{X}}_{\text{3}}-\text{8}.{\text{52X}}_{1}^{2}-3.14{\text{X}}_{2}^{2}-16.01{\text{X}}_{3}^{2} \end{array}$$

where Y_1_ is the inhibition, and X_1_, X_2_, X_3_ are independent variables in coded units. The regression model was designed using the F-test, and ANOVA was utilized to evaluate the significance and adequacy of the model (Raza et al., 2011).

As shown in Table 5, the *p*-values obtained using ANOVA and the F-test were < 0.01, demonstrating that the model terms were significant (*p* < 0.01). The coefficients for X_1_ and X_2_ were highly significant (*p* < 0.01), meaning that glucose and corn flour had a remarkable impact on the inhibition ability of postbiotics. The model was highly significant with a very low *p*-value < 0.01 (*p*-value Probability > F). The *R*^2^ and adjusted *R*^2^ values for inhibition were 0.9826 and 0.9670, respectively, which demonstrated the high accuracy of the polynomial regression model (Zhang et al., 2020).

**Table 5:** Analysis of CCD test results for inhibition.

**Source**	**df**	**Adj SS**	**Adj MS**	***F*-value**	***p*-value**
Model	9	5,835.98	648.44	62.86	< 0.0001
X_1_	1	228.42	228.42	22.14	0.0008
X_2_	1	518.16	518.16	50.23	< 0.0001
X_3_	1	18.11	18.11	1.76	0.2147
X_1_X_2_	1	244.98	244.98	23.75	0.0006
X_1_X_3_	1	19.56	19.56	1.90	0.1985
X_2_X_3_	1	400.02	400.02	38.78	< 0.0001
${\text{X}}_{1}^{2}$	1	1,045.72	1,045.72	101.37	< 0.0001
${\text{X}}_{2}^{2}$	1	141.85	141.85	13.75	0.0041
${\text{X}}_{3}^{2}$	1	3,695.62	3,695.62	358.26	< 0.0001
Residual	10	103.16	10.32		
Lack of fit	5	85.35	17.07	4.79	0.0552
Pure error	5	17.80	3.56		
Cor total	19	5,939.13			

R^2^ = 98.26%; adj R^2^ = 96.70%, df: degree of freedom; Adj SS: adjust the sum of squares; Adj MS: adjust mean square.

**Figure 3:**
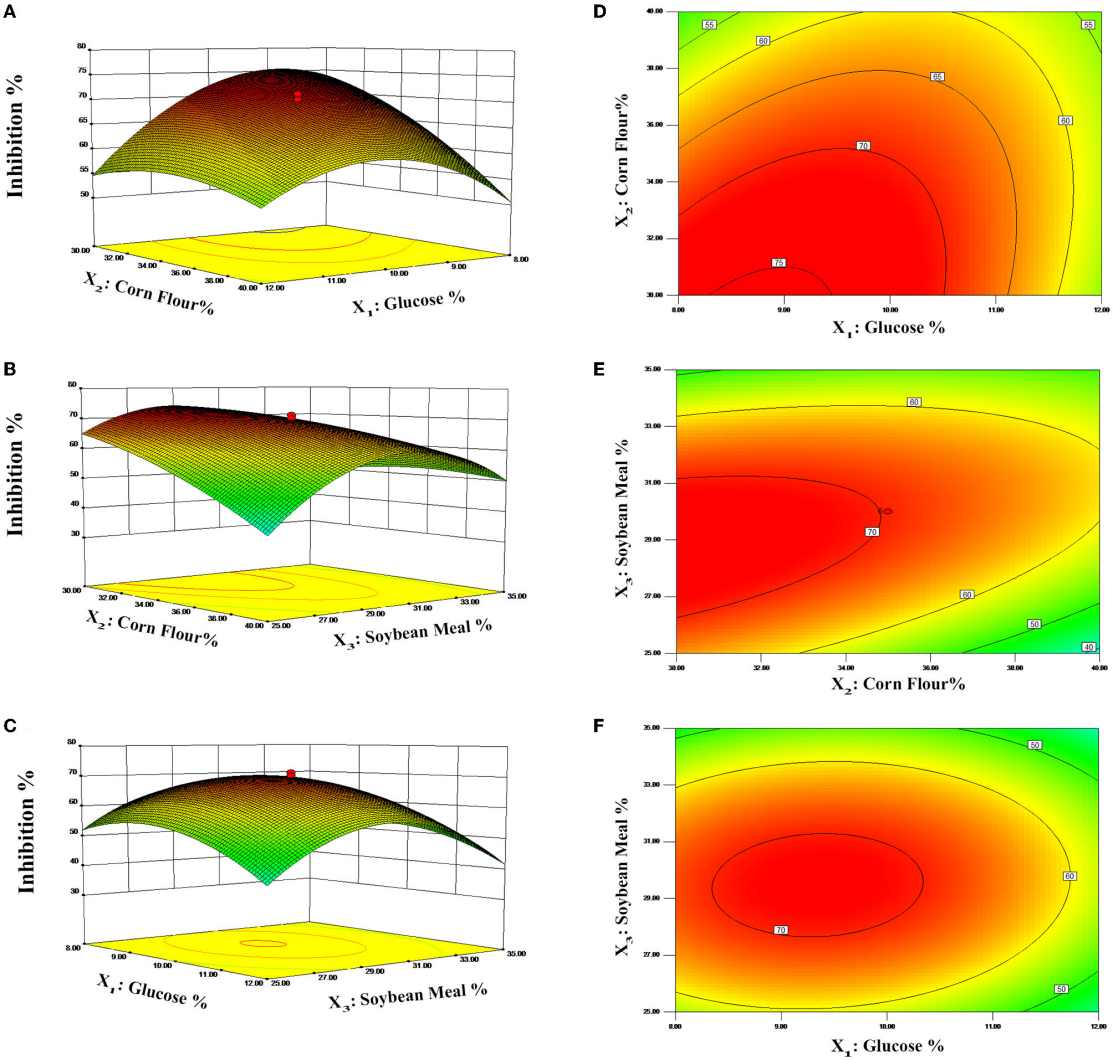
Response surface curves **(A–C)** and contour plots **(D–F)** predicted by the retained polynomial model.

The contour plots and 3D response surfaces to visually demonstrate the polynomial regression are shown in Figure 3. These findings also demonstrated the response across a range of independent variables and the association between the experimental levels of each factor. Figures 3A, D displays the impact of glucose and corn flour on antibacterial ability, and the maximum inhibition rate was observed when the addition of glucose and corn flour was at 8.82 and 30.02%, respectively. Subsequently, the inhibition rate of postbiotics decreased as the addition of corn flour and glucose increased, as presented in Figures 3B, E, C, F. With the increase of soybean meal by more than 30%, a decline in the inhibition rate was observed (Figures 3C, F). According to the model, the maximal value of the antimicrobial activity of 76.80% is achieved for X_1_ = 8.82, X_2_ = 30, and X_3_ = 28.57 (%). Finally, according to the optimized contents addition obtained from the CCD model, the experiment was repeated three times in fermentation bags to verify the real reliability of the predicted values. The inhibition rate was measured to be 78.56%, which was close to the predicted value. Compared with the initial antimicrobial activity (21.69%) of the fermentation condition, the optimized condition improved the inhibition against *E. coli* ATCC 25922 by 3.62 times.

### 3.5. Bioactive profiling of postbiotics

The profiles of bioactive contents of postbiotics are presented in Table 6, and the phenyllactic acid, lactic acid, acetic acid, butyric acid, pentanoic acid, total soluble sugar, and total phenolic acid including ferulic acid significantly increased (*p* < 0.05) by optimization process except for the total protein and propionic acid.

**Table 6:** Contents in postbiotics analyzed by colorimetric and modern instrument analytical methods.

**Contents**	**After optimization (mg/g)**	**Before optimization (mg/g)**	***p*-value**
Total protein	1.528 ± 0.0519	1.599 ± 0.0598	0.19271
Phenyllactic acid	0.178 ± 0.0275	0.07 ± 0.02	0.00528
Lactic acid	52.273 ± 0.5768	10.926 ± 0.1148	< 0.0001
Acetic acid	4.565 ± 0.0815	0.761 ± 0.0979	< 0.0001
Butyric acid	2.601 ± 0.3145	0.489 ± 0.0836	0.00036
Propionic acid	0.075 ± 0.0175	0.085 ± 0.0275	0.60858
Pentanoic acid	0.543 ± 0.0178	0.737 ± 0.0315	0.00075
Total Phenolic acid	4.951 ± 0.0377	2.332 ± 0.0057	< 0.0001
Ferulic acid	0.187 ± 0.0002	0.004 ± 0.0001	< 0.0001
Total soluble sugar	86.855 ± 3.3178	67.8791 ± 2.7364	0.00146

Data were given as the mean value ± SD from three biological replicates.

### 3.6. Determination of minimal inhibitory concentrations and minimal bactericidal concentrations

The MICs of postbiotics against all tested strains, including *S. aureus, S. typhimurium, S. pullorum, Escherichia coli, C. perfringens*, and *P. aeruginosa*, were within the range of 12.5–25 mg/mL, while the MBCs ranged from 12.5 to 50 mg/mL (Table 7). Postbiotics showed effective antibacterial activity (the lowest values of MICs with 12.5 mg/mL) against *S. aureus* ATCC 1882, *C. perfringens* CVCC 2030, *P. aeruginosa* ATCC 27853 and *P. aeruginosa* ATCC 9027 compared with other tested strains. As for the MASA strain, *S. aureus* ATCC 43300, postbiotics represented antibacterial ability with MIC of 18.75 mg/mL and MBC of 25 mg/mL, which showed the same results in *S. aureus* ATCC 6385 and *S. aureus* ATCC 25923. The MICs values of postbiotics recorded 25 mg/mL for *E. coli* ATCC 25922 and *E. coli* O157: H7 ATCC 43889 and the MBCs values displayed 50 and 43.75 mg/mL, respectively. *Escherichia coli* strains of ATCC 25922, ATCC 25325, and *E. coli* O157: H7 ATCC 43889 showed higher concentrations of MBCs compared to other indicator bacteria.

**Table 7:** MICs and MBCs of CTPs on different pathogens.

**Strains**	**MIC (mg/mL)**	**MBC (mg/mL)**
*S. aureus* ATCC 43300	18.75	25
*S. aureus* ATCC 6385	18.75	25
*S. aureus* ATCC 1882	12.5	25
*S. aureus* ATCC 25923	18.75	25
*S. typhimurium* ATCC 14028	18.75	31.25
*S. pullorum* CVCC 519	18.75	31.25
*E. coli* ATCC 25922	25	50
*E. coli* ATCC 25325	18.75	50
*E. coli* O157: H7 ATCC 43889	25	43.75
*C. perfringens* CVCC 2030	12.5	12.5
*P. aeruginosa* ATCC 27853	12.5	25
*P. aeruginosa* ATCC 9027	12.5	25

### 3.7. *In vivo* antioxidant activity of postbiotics

**Figure 4:**
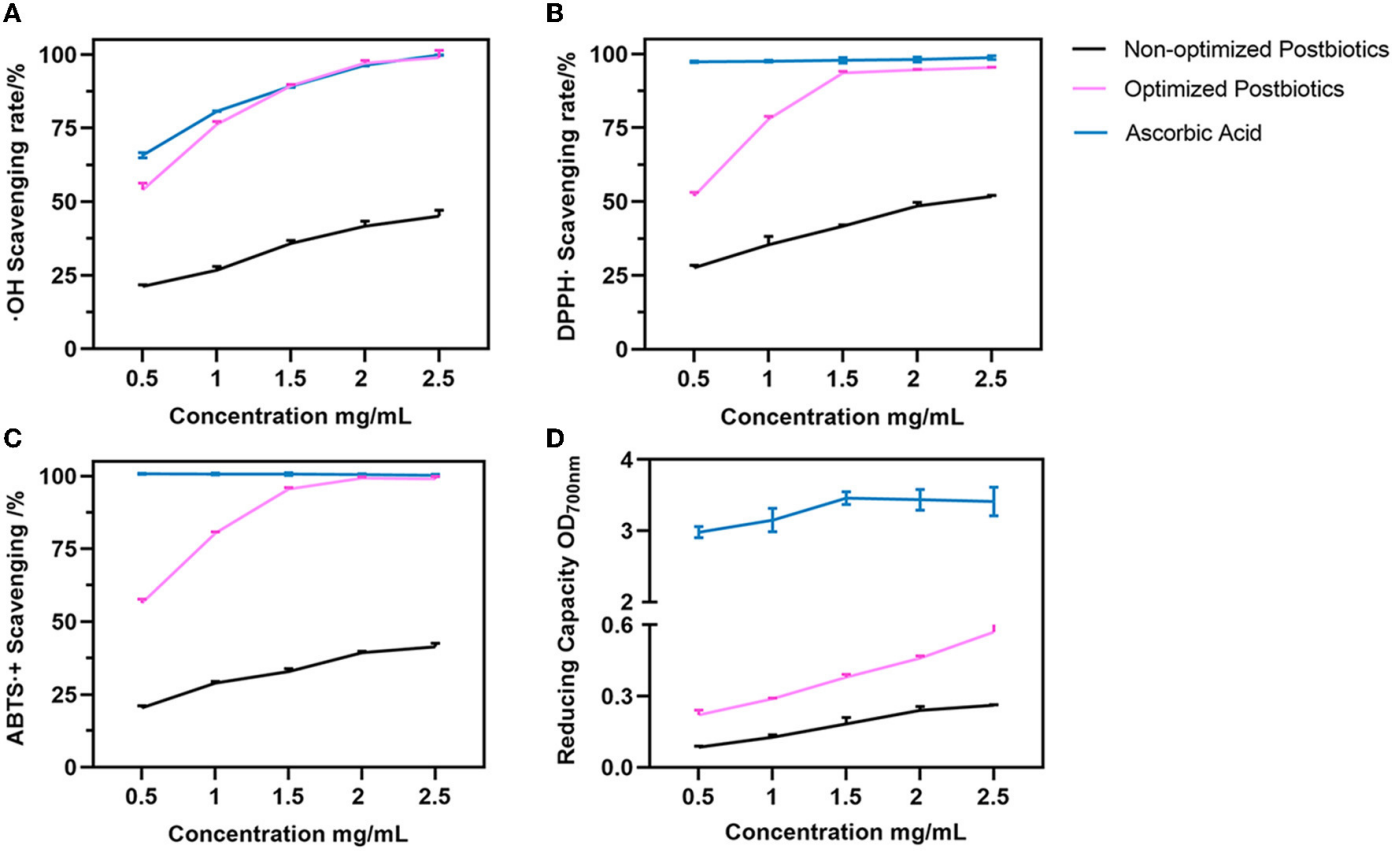
Hydroxyl radical **(A)**, DPPH **(B)**, ABTS **(C)**, and radical scavenging and reducing capacity **(D)** between the non-optimized and optimized postbiotics. Ascorbic acid as a positive control. Data were given as the mean value ± SD from three biological replicates.

In this study, the *in vitro* antioxidant activity of postbiotics was evaluated by hydroxyl radical, DPPH, and ABTS radical scavenging and reducing capacity. The results are shown in Figure 4. The IC_50_ value of postbiotics for hydroxyl radical, DPPH, and ABTS radical was ~0.5 mg/mL. However, the scavenging ability was lower than 50% even though the postbiotic concentration was higher before optimization. Optimized postbiotics presented superior activity to non-optimized postbiotics against hydroxyl radical, DPPH, and ABTS radical and better reducing capacity as shown in Figure 4. Optimized postbiotics showed similar activity to ascorbic acid in terms of hydroxyl, DPPH, and ABTS radical scavenging ability at a same concentration of 1.5–2 mg/mL. The reducing ability of postbiotics depended on the concentration.

### 3.8. Mammals' cell viability

**Figure 5:**
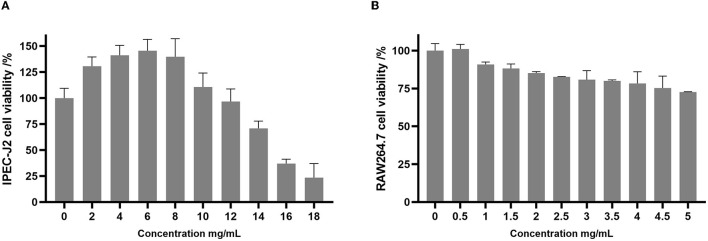
Cell viability of IPEC-J2 **(A)** and RAW264.7 **(B)** upon exposure to postbiotics. Data were given as the mean value ± SD from three biological replicates.

The cell viability of the intestinal porcine epithelial cell line (IPEC-J2) and the mouse macrophage cell line (RAW 264.7) was assessed after treatment with varying concentrations of postbiotics (Figures 5A, B). The results of the present study demonstrated that postbiotics did not affect the viability of IPEC-J2 cells when treated with concentrations up to 12 mg/mL. Concentrations of postbiotics ranging from 2 to 10 mg/mL even promoted the growth of IPEC-J2 cells (Figure 5A). On the other hand, RAW 264.7 macrophage cells exhibited lower tolerance to postbiotics, with cell viability being preserved at ~75% when treated with concentrations up to 5 mg/mL (Figure 5B).

### 3.9. Effect of postbiotics on NO production during the inflammatory response

**Figure 6:**
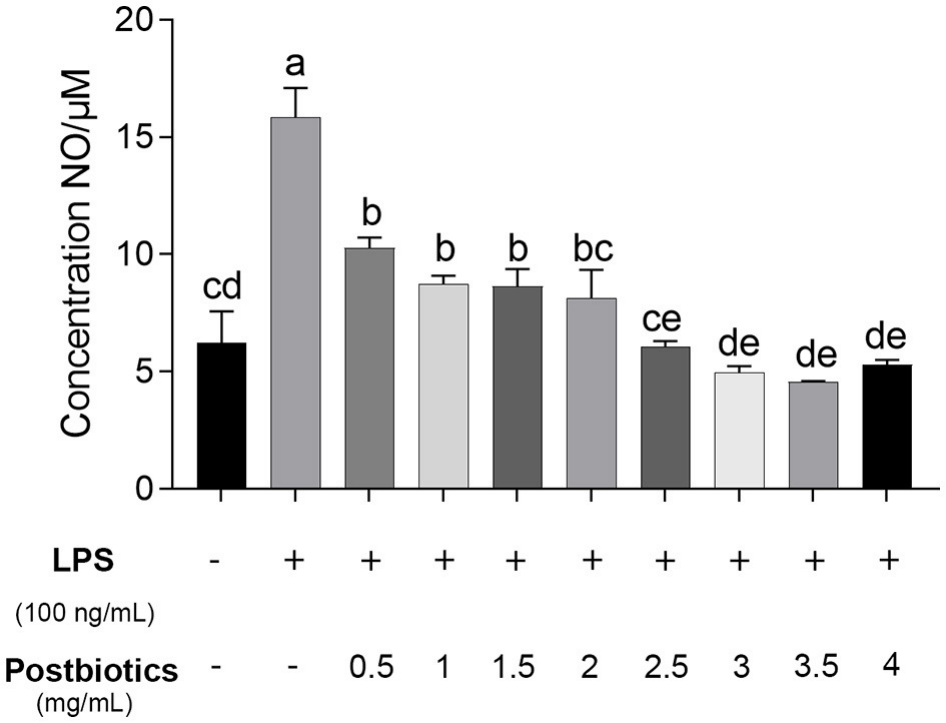
Effect of postbiotics on the production of NO in the LPS-induced RAW 264.7 cells. a–e: different lowercase letters show significance among different concentrations, and *p*-values of < 0.05 were expressed as mean ± SEM (*n* = 3).

As shown in Figure 6, NO production is significantly induced by lipopolysaccharide (LPS) at a concentration of 15.8 μM (*p* < 0.05). Postbiotics significantly inhibited NO production in LPS-activated RAW 264.7 macrophages starting at 0.5 mg/mL (*p* < 0.05), and 0.5–1.5 mg/mL of postbiotics reduced the same extent of NO content secreted by mouse macrophagocytes (*p* > 0.05). When the concentration of postbiotics was 2.0 mg/mL, the content of NO was decreased to the same level as the control group without LPS co-incubation. With the increase in the number of postbiotics to 4 mg/mL, the secretion of NO from RAW 264.7 cells also declined. Therefore, the minimum effective concentration of postbiotics to lessen NO production was 2 mg/mL. These results also reflected that the postbiotics showed anti-inflammatory ability against LPS-induced inflammation.

### 3.10. Wound healing capabilities of postbiotics *in vitro*

**Figure 7:**
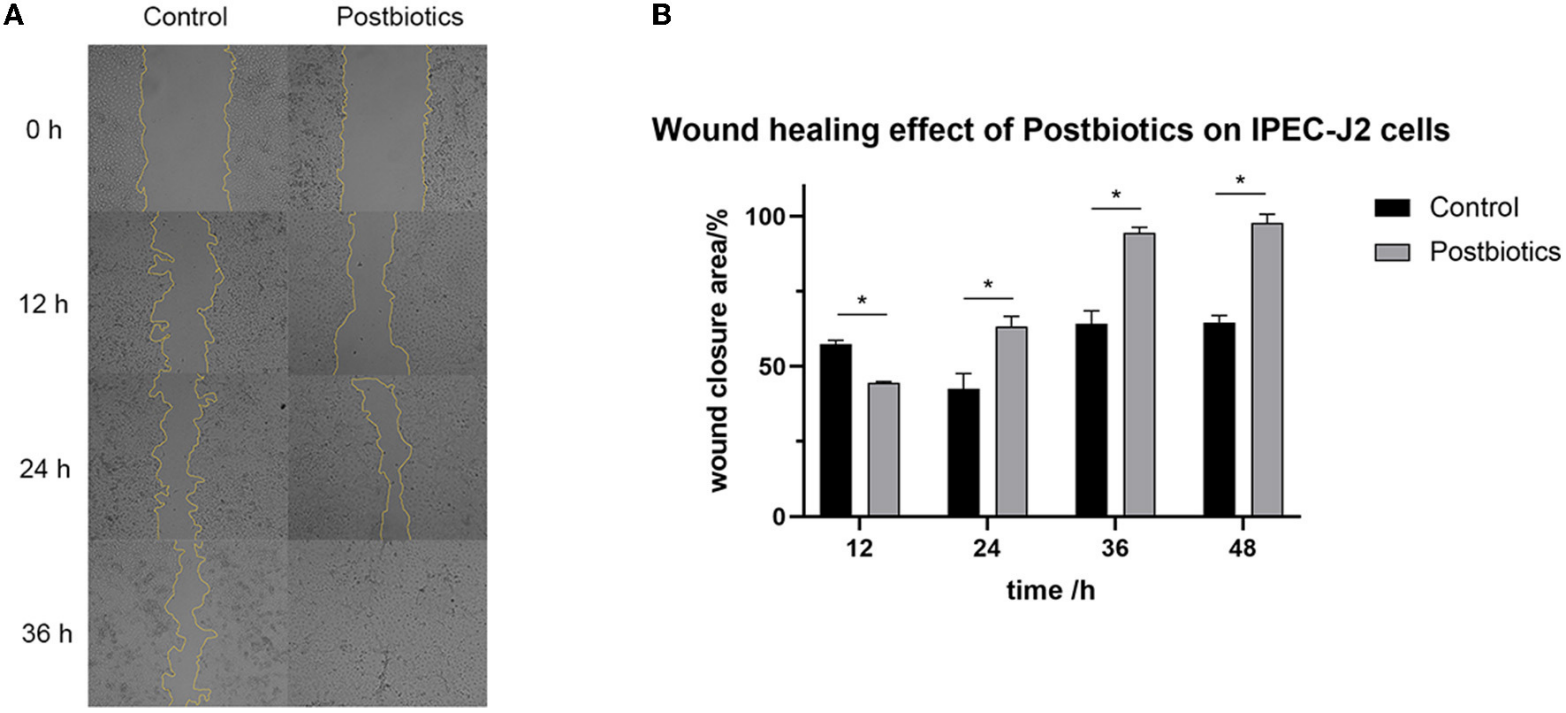
Impact of postbiotics (2.8 mg/mL) on the wound healing capacity of IPEC-J2 cells. **(A)** The state of cells in every 12 hours (magnification: 40 × ). **(B)** The descriptive results of wound closure area. *p < 0.05. Data were given as the mean value ± SD from three biological replicates.

Treatment of IPEC-J2 cells with postbiotics (2.8 mg/mL) after scratching led to a significant increase in wound healing when compared to the control (Figures 7A, B). In the first 12 h, treatment with 2.8 mg/mL of postbiotics did not provide any enhancement of the wound closure speed. In fact, the enhancement of the wound closure area in the postbiotic treatment was more than controlled after 24 h. Furthermore, complete healing was observed after 36 h of postbiotic treatment. As time extended to 48 h (images not shown), the control treatment also could not achieve complete healing.

## 4. Discussion

Scientific evidence has demonstrated that postbiotics exhibit diverse functional properties, which include, but are not limited to, antimicrobial, antioxidant, and immunomodulatory activities (Aguilar-Toalá et al., 2018; Cuevas-Gonzalez et al., 2020). Furthermore, postbiotics have great potential in the food industry as functional supplements, food quality improvers, and food preservatives (Zhong et al., 2022). In the present study, the antibacterial activity was increased by 3.62 times after the optimization of preparation conditions and fermentation medium. The profile of bioactive substances in postbiotics was also detected by colorimetric determination and modern instrument analytical methods. The optimized postbiotics showed a broad-spectrum antibacterial effect, antioxidant ability, and anti-inflammatory properties *in vitro*. We also evaluated the cell safety of postbiotics, which exhibited absolute safety at concentrations within the effective range. Furthermore, postbiotics had improved intestinal cell wound healing and gut health benefit potential.

The strain combinations with *B. amyloliquefaciens* J and *L. plantarum* SN4 tended to yield higher antibacterial ability compared to single fermentations of the J strain which was 35%, and SN4 was 67%, under the same conditions. *B. amyloliquefaciens* was found to secrete abundant amylase, protease, and cellulase (González Pereyra et al., 2020; Ngalimat et al., 2021), while *B. amyloliquefaciens* J exhibited strong abilities on amylase and protease, as shown in Supplementary Figure S2. On the other hand, *L. plantarum* SN4 exhibited weak activity of amylase and protease but could produce lactic acid and other bioactive substances from degradable carbon sources, which suggested that strain combination fermentation in SSF compensated for the incomplete enzyme systems of individual strains (Jia et al., 2019; Huang et al., 2023). The optimum temperature, water content, and inoculum size were 37°C, 50, and 8%, respectively, indicating that antimicrobial properties could be compromised by excessively high temperatures, too much water in fermentation progress, and living bacteria. It has been reported that appropriate temperature, water, and inoculum in solid media are crucial for creating an ideal environment for microorganisms' growth and metabolism (Guo et al., 2019). The fermentation time is determined by various factors such as the growth stage of bacteria strains, the production of inhibitors, and nutrient availability (Kanagasabai et al., 2019). Based on the findings of the one-factor-at-a-time experiments in this study, it was determined that a fermentation time of 8 days was appropriate. The optimized fermentation conditions of postbiotic preparation greatly enhanced the inhibition rate against *E. coli* ATCC 25922 in the initial stage of optimization.

To maximize the antimicrobial ability of postbiotics, Plackett–Burman design (PBD) and central composite design (CCD) of response surface methodology (RSM) were applied to optimize the fermentation medium composition. Wang et al. (2017) reported an increase of 3.8 times in antimicrobial activity with optimal medium composition compared with the initial medium. Ghribi et al. (2012) proved that a central composite design and a response surface methodology successfully increased the antimicrobial activity of biosurfactants. PBD and RSM had become common strategies to increase the production of bactericidal substances in SSF but were barely used in postbiotic preparation (Yun et al., 2018; Chen et al., 2021). In this study, corn flour, soybean meal, and glucose had a significant positive effect on antibacterial ability (*p* < 0.05). These three significant factors, according to the PBD results were, adjacently optimized by the steepest ascent method (SAM), and the optimal point was further used as the center point in the CCD. Based on the solution of polynomial regression of predicted responses and validation experiments, the inhibition rate for *E. coli* ATCC 25922 was found to have increased by 3.62 times compared to the original medium. By combining the results of fermentation conditions optimization, we have developed postbiotics with significantly higher bioactivities using agriculture by-products such as corn flour, soybean meal, and bran powder. Moreover, 3-phenyllactic acid, lactic acid, acetic acid, butyric acid, pentanoic acid, total phenolic acid, and total soluble sugar were significantly increased compared to unoptimized conditions (*p* < 0.05).

The postbiotics prepared in this study demonstrated broad-spectrum antibacterial properties against both gram-positive and gram-negative bacteria. It was revealed that 3-phenyllactic acid, lactic acid, and acetic acid might be the key points. The 3-phenyllactic acid was reported to possess a broad-spectrum antibacterial activity linked to severely affecting the structure of biomembranes (Kleinwachter et al., 2021; Jiang et al., 2022). The organic acid was early reported to be used in controlling microbial contamination of carcass meat, attributing its antibacterial activity to it (Cherrington et al., 1991). In addition to antibacterial activity, postbiotics also demonstrated strong free radical scavenging ability and anti-inflammatory effects in the mouse macrophage cell line (RAW264.7).

Hydroxyl free radicals (OH·) and nitric oxide (NO·) are common examples of free radicals. The optimized postbiotics showed an IC_50_ value of 0.5 mg/mL to scavenge them, indicating that postbiotics had excellent antioxidant activity. The results obtained in this study were consistent with previous studies that reported the antioxidant properties of postbiotics derived from *L. plantarum* using liquid fermentation (Yu et al., 2016; Chang et al., 2021). Postbiotic RG14 (cell-free supernatant) produced by *L. plantarum* RG14 showed the highest antioxidant activity against ABTS and DPPH radicals and also enhanced the glutathione peroxidase (GPX) enzymes *in vivo* (Izuddin et al., 2020). In this study, optimized postbiotics exhibited an increased free radical scavenging ability against OH·, DPPH, and ABTS and a better reducing capacity compared to non-optimized postbiotics. Our results align with previous studies showing that optimizing fermentation conditions with response surface methodology significantly enhanced antioxidant activities (Miao et al., 2020; Chen et al., 2021; Kuo et al., 2021). Moreover, studies also revealed that the difference in the antioxidant activity of postbiotics depends on mechanisms such as the metal ion chelating ability, the antioxidant enzyme system, and the antioxidant metabolites present in the postbiotic (Yang et al., 2017). Polyphenols, including phenolic acids and fatty aromatic acids, contribute to the antioxidant activity of the postbiotics (Nikmaram et al., 2018). Incili et al. (2022) characterized postbiotics from lactic acid bacteria and found that the concentration and types of phenolics varied greatly, mainly depending on the medium composition. In our study, ferulic acid and total phenolic acid were significantly increased by optimization, which proved the influence of fermentation conditions on postbiotic bioactive components (*p* < 0.05). Additionally, the total soluble sugar in postbiotics was derived from bacterial metabolites, and degraded substrates such as exopolysaccharides and oligosaccharides are also conducive to antioxidant activity. The postbiotic characterization showed that the postbiotics had antioxidant properties that might be correlated with total phenolic acid, including ferulic acid and total soluble sugar in this research. Moreover, the detected bioactive components were also connected with anti-inflammatory capacity.

Lipopolysaccharide (LPS) is a common endotoxin derived from gram-negative bacteria that causes a series of inflammatory reactions in the body. Among these, NO is excessively generated by one of the pro-inflammatory enzymes, iNOS, and consequently results in diverse diseases (Patel et al., 1999). Our study indicated that the postbiotics possessed potent NO inhibitory activity against LPS-induced NO release with an effective concentration of 2 mg/mL in RAW264.7 cells. Meanwhile, to determine whether the inhibitory effects of compounds on NO production were due to specific anti-inflammatory activities, or non-specific cell cytotoxicity leading to false positive results, RAW 264.7 cell viability was determined by using CCK-8 assays. Notably, at a concentration of 5 mg/mL, no significant cytotoxic effects were observed. Normal NO production in the phagocytes is beneficial for host defense against pathogens and cancer cells. Recently, there have been a few studies on the anti-inflammatory activity of postbiotics. Kang et al. (2021) observed that lactic acid bacteria in heat-killed cells had decreased nitric oxide production via the downregulation of inducible nitric oxide synthase. In another study, lyophilized cell-free supernatants decreased the production of nitric oxide and were not cytotoxic to RAW 264.7 cells (Sornsenee et al., 2021).

In this study, we prepared postbiotics through simultaneous solid-state fermentation of *B. amyloliquefaciens* and *L. plantarum* for the first time and obtained postbiotics with antibacterial, antioxidant, and anti-inflammatory properties. More interestingly results in the cell viability test, postbiotics showed high cell safety in the intestinal porcine epithelial cell line (IPEC-J2) and even promoted the growth of the IPEC-J2 cells (Figure 5B). The mechanical barrier of the intestine is an important component of the intestinal barrier and plays a crucial role in maintaining intestinal health. Mechanical intestinal injuries were frequently happening in the body. Continuous production, migration, and apoptosis of epithelial cells provide a dynamic barrier for intestinal health. Previous studies in soluble proteins produced by probiotic bacteria promoted cell growth in human and mouse colon epithelial cells (Yan et al., 2007), and such probiotic-derived factors that bring beneficial effects had been identified as “postbiotic” mediators until 2012 (Tsilingiri et al., 2012). Postbiotics, such as lactobacilli-derived factors, have been proposed and described to enhance innate immunity, promote intestinal epithelial cell survival, and improve barrier function (Cicenia et al., 2014). In this study, we detected that the postbiotics enhance intestinal epithelial wound healing after a mechanical injury such as a cell scratch in IPEC-J2 cells. It was also reported by Lee et al. (2022) that the postbiotics of *L. reuteri* DS0384, prepared with liquid fermentation, promoted intestinal stem cell proliferation and protected intestinal epithelial cells from cytokine-induced injury. These findings indicated that postbiotics played a crucial role in health and nutrition.

## 5. Conclusion

This study has taken a novel approach for the optimized production of postbiotics through solid-state fermentation techniques, which significantly improved the antibacterial, antioxidant, and anti-inflammatory activities (*p* < 0.05). Furthermore, our findings suggest that the optimized postbiotics produced in our study may promote gut health by reducing inflammation, promoting intestinal epithelial growth, and protecting from mechanical injury. The current study represents postbiotics as a potential remedy for food preservatives and for promoting gut health. Further research is needed to explore the mechanism of action of these postbiotics and evaluate their pharmacological efficacy as drugs and food functional additives.

## Data availability statement

The datasets presented in this study can be found in online repositories. The names of the repository/repositories and accession number(s) can be found in the article/Supplementary material.

## Author contributions

YT conceived and designed the experiments. YT, HG, JZ, TY, and JL performed the experiments. YT, SP, and QC analyzed the data. TB, JW, YZ, and XW finished the visualization of the figures. YT wrote the manuscript. ZA corrected grammar errors. DS and ZA reviewed and edited the manuscript. RZ guided the experiments. All authors have read and agreed to the published version of the manuscript. All authors contributed to the article and approved the submitted version.
