# Supplementary material for: Optimizing postbiotic production through solid-state fermentation with Bacillus amyloliquefaciens J and Lactiplantibacillus plantarum SN4 enhances antibacterial, antioxidant, and anti-inflammatory activities
Source: Front Microbiol. 2023 Sep 7;14:1229952. doi: 10.3389/fmicb.2023.1229952 (PMC10512978; doi:10.3389/fmicb.2023.1229952)
Supplement: Supplementary file 1 [file Data_Sheet_1.docx]

Supplementary Material

Optimizing Postbiotic Production through Solid-State Fermentation with Bacillus amyloliquefaciens J and Lactiplantibacillus plantarum SN4 Enhances Antibacterial, Antioxidant, and Anti-inflammatory Activities

**Yucui Tong^1^, He’nan Guo^2^, Zaheer Abbas^1^, Jing Zhang^1^, Junyong Wang^1^, Qiang Cheng^1^, Shuyue Peng^1^, Tiantian Yang^1^, Ting Bai^1^, Yichen Zhou^1^, Jinzhuan Li^1^, Xubiao Wei^2^, Dayong Si^1^, Rijun Zhang*^1^.**

^1^ Laboratory of Feed Biotechnology, State Key Laboratory of Animal Nutrition, College of Animal Science and Technology, China Agricultural University, Beijing 100193, China;

^2^ School of Pharmaceutical Sciences, Tsinghua University, Beijing 100084, China;

*** Correspondence:** Corresponding Author: zhangrj621@126.com

# Supplementary Figures

##
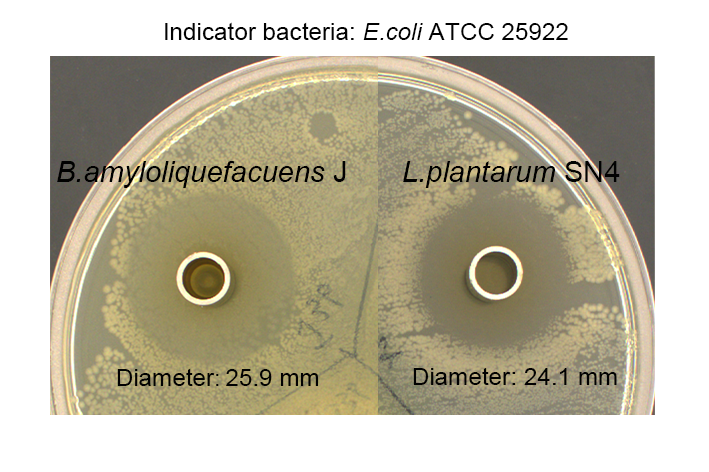
Supplementary Figures

Figure S1. The antibacterial activity of *Bacillus amyloliquefacuens* J and *Lactiplantibacillus plantarum* SN4 against *E.coli* ATCC 25922.
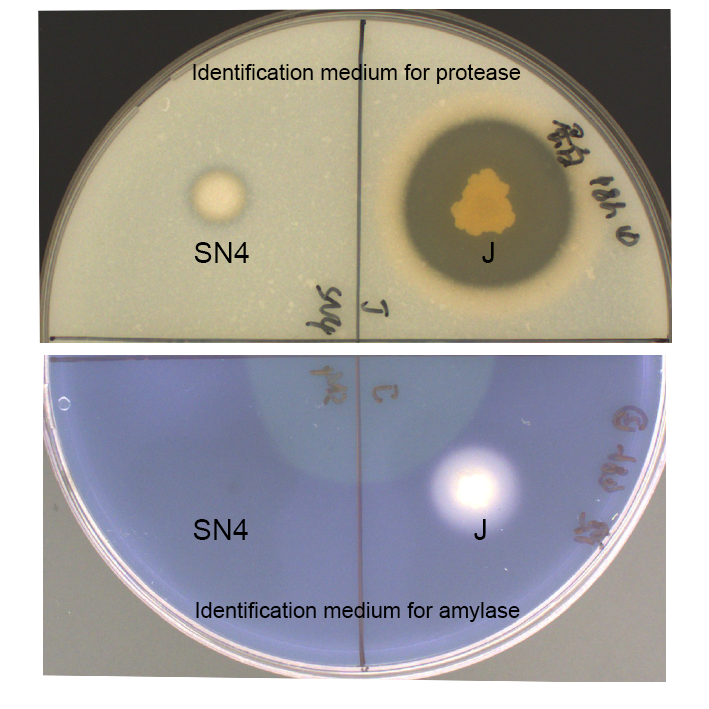


Figure S2. The protease and amylase production ability of *L. plantarum* SN4 and *B. amyloliquefacuens* J.
